# Foot and ankle problems in children and young people: a population-based cohort study

**DOI:** 10.1007/s00431-024-05590-8

**Published:** 2024-05-09

**Authors:** Emma Rezel-Potts, Catherine Bowen, Kate M. Dunn, Christopher I. Jones, Martin C. Gulliford, Stewart C. Morrison

**Affiliations:** 1 School of Life Course and Population Sciences, King’s College London, Addison House, Guy’s Campus, London, SE1 1UL UK; 2 Faculty of Environmental and Life Sciences, University of Southampton, Highfield Campus, Southampton, SO17 1BJ UK; 3 School of Medicine, Centre for Musculoskeletal Health Research, Keele University, Keele, Staffordshire, ST5 5BG UK; 4 Department of Primary Care and Public Health, Brighton and Sussex Medical School, Falmer, BN1 9PS UK

**Keywords:** Foot, Ankle, Epidemiology, CPRD

## Abstract

The aim of this research was to describe the epidemiology, presentation and healthcare use in primary care for foot and ankle problems in children and young people (CYP) across England. We undertook a population-based cohort study using data from the Clinical Practice Research Datalink Aurum database, a database of anonymised electronic health records from general practices across England. Data was accessed for all CYP aged 0–18 years presenting to their general practitioner between January 2015 and December 2021 with a foot and/or ankle problem. Consultation rates were calculated and used to estimate numbers of consultations in an average practice. Hierarchical Poisson regression estimated relative rates of consultations across sociodemographic groups and logistic regression evaluated factors associated with repeat consultations. A total of 416,137 patients had 687,753 foot and ankle events, of which the majority were categorised as “musculoskeletal” (34%) and “unspecified pain” (21%). Rates peaked at 601 consultations per 10,000 patient-years among males aged 10–14 years in 2018. An average practice might observe 132 (95% CI 110 to 155) consultations annually. Odds for repeat consultations were higher among those with pre-existing diagnoses including juvenile arthritis (OR 1.73, 95% CI 1.48 to 2.03).

*    Conclusions*: Consultations for foot and ankle problems were high among CYP, particularly males aged 10 to 14 years. These data can inform service provision to ensure CYP access appropriate health professionals for accurate diagnosis and treatment.

**Table:** 

**What is Known:** *• Foot and ankle problems can have considerable impact on health-related quality of life in children and young people (CYP).* *• There is limited data describing the nature and frequency of foot and ankle problems in CYP.*	
**What is New:** *• Foot and ankle consultations were higher in English general practice among CYP aged 10 to 14 years compared to other age groups, and higher among males compared to females.* *• The high proportion of unspecified diagnoses and repeat consultations suggests there is need for greater integration between general practice and allied health professionals in community-based healthcare settings.*

**Supplementary Information:**

The online version contains supplementary material available at 10.1007/s00431-024-05590-8.

## Introduction

Foot and ankle problems are common among children and young people (CYP) [1, 2] and can develop at any stage of the life course [1]. These problems have been shown to impact on many aspects of the lives of CYP, such as school attendance and participation in sport [3], and can be distressing for parents too [4]. The needs of CYP presenting to healthcare services with foot and ankle problems are diverse but poorly described in the literature; a recent analysis of the Australian Bettering the Evaluation and Care of Health program dataset [2] identified that injury, infection and dermatological conditions were the most frequently managed foot, ankle and leg problems in children. UK-based epidemiological studies are relatively scarce [1, 5] but demand for healthcare services for foot and ankle problems in CYP has been reported to be substantial [1].

Strategies to ensure that the impact of foot and ankle problems are mitigated through access to appropriate healthcare services are fundamental to supporting healthy musculoskeletal development [6], addressing inequalities [2] and supporting longer-term health and wellbeing for all CYP. This is particularly important for children with health conditions where care needs are often high and functional impairment secondary to foot and ankle problems are common [7–12]. As such, timely access to healthcare professionals and joined-up clinical services are key to the early detection of problems and reduction in adverse outcomes [9], such as long-term disability and chronic pain [2]. It is recognised that clinical services for foot and ankle problems in CYP are underdeveloped [1] and further research to understand the patterns of foot and ankle problems in CYP, and the factors that influence primary care access, is needed to influence service planning and allocation of resources. The aim of this research is to describe the epidemiology, presentation and healthcare use in primary care for foot and ankle problems in CYP across England.

## Methods

### Study population and data sources

The data source was the Clinical Practice Research Datalink (CPRD) Aurum, a database of anonymised electronic health records from general practices in England. It contains comprehensive medical record data, including prescriptions and clinical diagnoses. There are a total of 1491 contributing general practices in England with approximately 41 million currently registered patients in the May 2022 release [13]. There were 7,612,087 (52%) CYP from all English practices in the May 2022 release. Each patient has a unique anonymised numerical identifier enabling tracking through successive releases. CPRD Aurum is representative of the general population in terms of geographical distribution, deprivation, age and gender (category terminology as specified by CPRD) [14]. Linked socioeconomic data from the Index of Multiple Deprivation (IMD) for patient postcode and practice postcode, and secondary care data from Hospital Episode Statistics, were provided by CPRD. Approximately 75% of CPRD practices in England are eligible for linkage. Where CPRD ethnicity data were missing, we used available Hospital Episode Statistics ethnicity data. The study protocol was reviewed via Research Data Governance Process and approved by the CPRD team (protocol number 20_ 002137). CPRD has ethical approval from the Health Research Authority to support research using anonymised patient data. All work was conducted in accordance with the Declaration of Helsinki.

We extracted data for all CYP up to the age of 18 years between the 1st of January 2015 and 31st of December 2018 with any foot and ankle coded events during the period 1st of January 2015 to 31st of December 2021 in the May 2022 release. We excluded patients from practices in Northern Ireland, Scotland or unknown regions (also excluded from the CPRD denominator file for rate calculations). There were no exclusion criteria related to demographic, clinical or geographic characteristics.

### Main measures

The cohort was selected based on any recorded foot and ankle event (with a maximum age of 18 years at index date) in the study period using Systematized Nomenclature of Medicine Clinical Terms (SNOMED CT). These were grouped into code categories derived from existing research [1]. Covariates were defined using data recorded in the study period before the index date. Covariates were selected because of known associations with foot and ankle problems and included ethnicity, IMD for practice and patient, age category, gender (male or female—covariate and category terminology as specified by CPRD) [15], further categories of gender excluded due to low counts (< 0.001%)), region of practice, pre-existing health diagnoses and body mass index (BMI). The BMI values were converted to *z*-scores and adjusted for age and gender using the British 1990 growth reference data population [16]. Social deprivation data were derived from participant postal code of residence and practice postal code based on IMD 2019 classification at lower super output area, divided into quintiles based on the national distribution from the first quintile (most deprived) to the fifth quintile (least deprived) [17].

### Analysis

Person-time at risk (“patient-years”) was calculated using the May 2022 CPRD Aurum denominator file to identify individual registration time for all eligible patients from practices in the sample and aggregated by gender, age group and year. We calculated age- and gender-specific rates of foot and ankle consultations per 10,000 patient-years. Adjusted and unadjusted hierarchical Poisson regression models with patient-years as offset and practice identifier as a random effects variable were fitted to estimate the relative rate of foot and ankle consultations according to gender (with males as the reference group), age group (with 10 to 14 years as the reference group), year of diagnosis (with 2015 as reference) and region (with South East of England as reference). Hierarchical multivariable logistic regression analysis using binomial distribution and a logit link function evaluated sociodemographic associations and pre-existing diagnoses with repeat consultations within 6 months. Included in the model were gender, age category, ethnic group, practice IMD and pre-existing diagnoses and with practice identifier as a random effects variable. Subgroup analyses evaluated associations between these factors and repeat consultations for categories of codes musculoskeletal, dermatological, unspecified pain and infection. Repeat consultation was defined as a foot and ankle coded event on a different date within 6 months of the index event. A directed acyclic graph was constructed to depict assumed relationships between the exposure and outcome and all variables included in the fully adjusted analysis model. We calculated the number of events expected (and the 95% confidence intervals (CIs)) among CYP in a general practice with 10,000 patients (the general practice mean list size for England) during the study period, but for this analysis, years 2020 and 2021 were excluded as these years had highly unusual attendance rates due to the COVID-19 pandemic. We used the average consultation rates calculated for our study population during 2015 to 2019 to estimate expected numbers of consultations for all foot and ankle health events, for subcategories of foot and health codes and for the top ten individual codes among numbers of CYP for this average practice. Analyses were performed using R version 4.2.3 [18]. The “stats” package [19] was used for analysis, and “ggplot2” [20] and “forestplot” [21] were used to construct plots. The DAG was constructed using DAGitty version 3.1 [22].

## Results

### Characteristics of study population

Among the 7,612,087 patients under 18 years in CPRD Aurum, there were 416,137 patients with 687,753 coded events for foot and ankle problems from 1st of January 2015 to 31st of December 2021 from 1448 practices (see online resource 1). Descriptive characteristics for the cohort and their total number of coded events are presented in Table 1. The mean age of the study population was 10.7 years (standard deviation, 4.6), and the age category with the highest frequency of first (42%) and total (44%) primary care events was 10 to 14 years. There were more males (52%) than females (48%) in the cohort and across all the age categories apart from category 5 to 9 years where 70,090 (53%) were females compared to 62,087 (47%) males. Most participants (67%) had only one coded event during the study period. The most common category was “musculoskeletal” (34%), followed by “unspecified pain” (22%), “dermatological” (21%) and “infection” (11%). Participants were mostly in the white ethnic group (77%), followed by Asian (7%). Based on the indices of multiple deprivation, slightly more practices were categorised in the least deprived regions (24%). The most frequently recorded pre-existing diagnoses were autism (4%) and ADHD (4%), although those with intellectual disability had the highest proportion of total coded events (5%). Most participants did not have a BMI (*z*) recording in the years pre and post their index date or were below the age of 3 during this recording (90%). Whilst the BMI (*z*) was unknown for most of the total coded events (42%), there were more BMI (*z*) records overall, indicating that over a third of consultations were with patients with a normal BMI (*z*), 13% overweight and 11% obese.

**Table 1:** Cohort characteristics and outcome frequencies. Figures are frequencies (column percent) except where indicated

	**Patients**	**Coded events**
**Total**	416,137 (100)	687,753 (100)
**No. of coded events**		
One	278,443 (67)	-
Two	78,883 (19)	-
Three to five	49,345 (12)	-
Six to ten	8156 (2)	-
More than ten	1310 (0)	-
Foot and ankle code category*		
Musculoskeletal	153,701 (37)	236,880 (34)
Unspecified pain	93,596 (22)	148,137 (22)
Dermatological	72,344 (17)	143,575 (21)
Infection	45,878 (11)	76,315 (11)
Fracture	31,755 (8)	52,484 (8)
Miscellaneous	17,088 (4)	25,879 (4)
Surgical procedure	1494 (0)	3956 (1)
Nerve	224 (0)	436 (0)
Tumour	36 (0)	59 (0)
Circulatory issue	21 (0)	32 (0)
**Age group (years)**		
0 to 4	54,260 (13)	72,787 (11)
5 to 9	92,802 (22)	132,177 (19)
10 to 14	173,823 (42)	300,272 (44)
15 to 18	95,252 (23)	182,517 (27)
**Gender**		
Male	218,065 (52)	361,639 (53)
Female	198,072 (48)	326,114 (47)
Body mass index (*z*)**		
Normal weight	27,652 (7)	235,535 (34)
Overweight	9036 (2)	85,970 (13)
Obese	6717 (2)	74,039 (11)
Unknown	372,732 (90)	292,209 (42)
**Ethnic group**		
White	319,115 (77)	540,159 (53)
Asian	29,231 (7)	44,779 (7)
Black	17,522 (4)	26,589 (4)
Mixed	14,543 (3)	22,390 (3)
Other	14,965 (4)	22,539 (3)
Not known	20,761 (5)	31,297 (5)
**IMD (practice)**		
First quintile (most deprived)	73,027 (18)	119,895 (17)
Second quintile	70,079 (17)	117,308 (17)
Third quintile	85,164 (20)	143,441 (21)
Fourth quintile	88,136 (21)	145,194 (21)
Fifth quintile (least deprived)	99,731 (24)	161,915 (24)
**IMD (patient)**		
First quintile (most deprived)	87,557 (21)	146,951 (21)
Second quintile	79,024 (19)	131,047 (19)
Third quintile	75,054 (18)	126,060 (18)
Fourth quintile	79,410 (19)	129,229 (19)
Fifth quintile (least deprived)	88,238 (21)	143,988 (21)
Unknown	6854 (2)	10,478 (2)
**Region**		
South East	89,307 (21)	146,230 (21)
North West	80,234 (19)	133,886 (19)
South West	71,161 (17)	92,956 (14)
West Midlands	68,077 (16)	114,247 (17)
London	54,243 (13)	107,314 (16)
East of England	20,574 (5)	33,820 (5)
East Midlands	7033 (2)	12,078 (2)
North East	14,561 (3)	23,918 (3)
Yorkshire and the Humber	12,982 (3)	23,304 (3)
**Pre-existing diagnoses**		
Lupus	100 (0)	169 (0)
Juvenile arthritis	766 (0)	1607 (0)
Intellectual disability	7827 (2)	14,427 (5)
Diabetes	6536 (2)	11,719 (2)
Autism	14,870 (4)	27,186 (4)
Cerebral Palsy	1261 (0)	2485 (0)
ADHD	11,260 (3)	20,084 (3)

*IMD* Index of Multiple Deprivation, *ADHD* attention-deficit hyperactivity disorder

*This is the category of code for the first coded event per patient in the study period for column, “Patients”; **Z-scores using BMI measures in the year prior or after the index date or date of any consultation and not including measures recorded at ages below 3 years

The frequency of the ten most recorded foot and ankle consultation codes are shown in Table 2—“ingrowing great toenail” was observed 110,624, representing 16% of total codes, followed by “foot pain” (10%) and “paronychia of toe” (7%). The frequency of the ten most recorded foot and ankle events for males and females is reported (see online resource 2).

**Table 2:** Frequency of the ten most commonly recorded foot and ankle consultation codes

**Code Description**	**Frequency (%)**^**a**^
Ingrowing great toenail	110,624 (16)
Foot pain	66,059 (10)
Paronychia of toe	45,009 (7)
Ankle sprain	43,477 (6)
Ankle pain	38,965 (6)
Ankle injury	35,022 (5)
Foot injury	25,691 (4)
Injury of toe	19,958 (3)
Heel pain	17,987 (3)
Flat foot	16,446 (2)

^a^Percentage of overall code total, *n* = 687,753

**Fig. 1:**
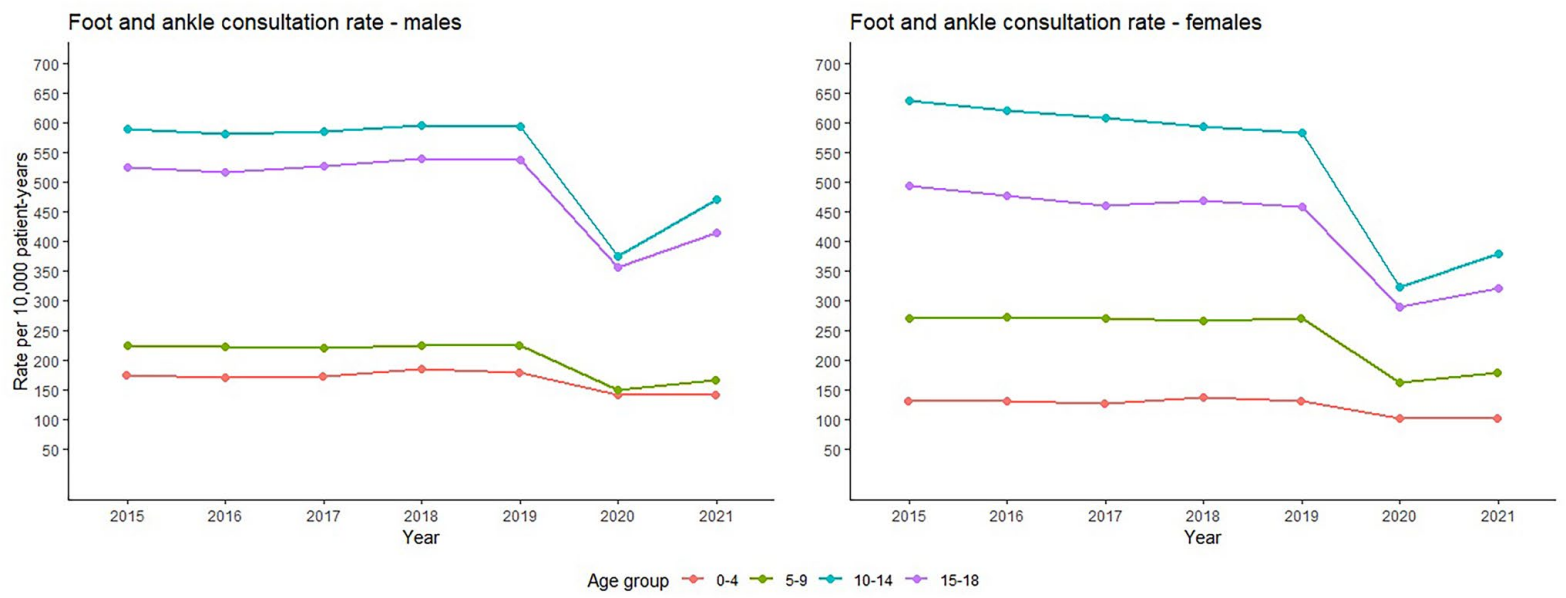
Rate per 10,000 patient-years of foot and ankle consultations in CPRD (2015 to 2020)

Rates of foot and ankle health consultations peaked at 601 consultations per 10,000 patient-years among males aged 10 to 14 years in 2018 and 641 consultations per 10,000 patient-years among females aged 10 to 14 years in 2015 (Fig. 1). The average rate across the study period was 343 (SD = 178) per 10,000 patient-years overall and 352 (SD = 179) and 333 (SD = 179) for males and females, respectively.

Table 3 shows incident rate ratios (95% confidence intervals) for foot and ankle consultations, unadjusted and adjusted for age group, gender, year and region. Being female was associated with lower consultation rates for foot and ankle health than being male (adjusted rate ratio (ARR) 0.96; 95% CI 0.95 to 0.96). Most regions were associated with a higher rate of consultations compared to the South East, apart from London, which was associated with a lower rate (ARR 0.74; 95% CI 0.68 to 0.81). Years 2020 and 2021 were associated with lower rates compared to the 2015 reference year: 2020 (ARR 0.62; 95% CI 0.61 to 0.63) and 2021 (ARR 0.71; 0.70 to 0.72).

**Table 3:** Poisson regression analysis showing unadjusted and adjusted relative rates for cohort characteristics

**Variable**	**Unadjusted** RR (LL UL)	**Adjusted**^**a**^ RR (LL UL)
Gender		
Male	Ref.	Ref.
Female	0.95 (0.94 to 0.96)	0.96 (0.95 to 0.96)
Age group		
0 to 5	0.28 (0.27 to 0.28)	0.28 (0.27 to 0.28)
6 to 9	0.42 (0.42 to 0.43)	0.42 (0.41 to 0.43)
10 to 14	Ref	Ref
15 to 18	0.84 (0.83 to 0.85)	0.84 (0.84 to 0.85)
Region		
South East	Ref.	Ref.
East Midlands	1.09 (0.91 to 1.31)	1.10 (0.91 to 1.31)
East of England	1.20 (1.03 to 1.40)	1.21 (1.03 to 1.41)
London	0.72 (0.66 to 0.78)	0.74 (0.68 to 0.81)
North East	1.26 (1.09 to 1.45)	1.27 (1.10 to 1.46)
North West	1.06 (0.98 to 1.15)	1.08 (0.99 to 1.17)
South West	1.25 (1.14 to 1.38)	1.25 (1.13 to 1.39)
West Midlands	0.99 (0.91 to 1.08)	0.99 (0.91 to 1.08)
Yorkshire and the Humber	1.12 (0.95 to 1.31)	1.09 (0.93 to 1.28)
Year		
2015	Ref.	Ref.
2016	0.99 (0.98 to 1.01)	0.98 (0.97 to 1.00)
2017	1.00 (0.98 to 1.01)	0.98 (0.97 to 0.99)
2018	1.02 (1.00 to 1.04)	0.99 (0.98 to 1.00)
2019	1.02 (1.00 to 1.03)	0.98 (0.97 to 0.99)
2020	0.65 (0.64 to 0.66)	0.62 (0.61 to 0.63)
2021	0.75 (0.74 to 0.77)	0.71 (0.70 to 0.72)

^a^Model was adjusted for age, gender, region and year and included a random effect to account for clustering by practice

**Fig. 2:**
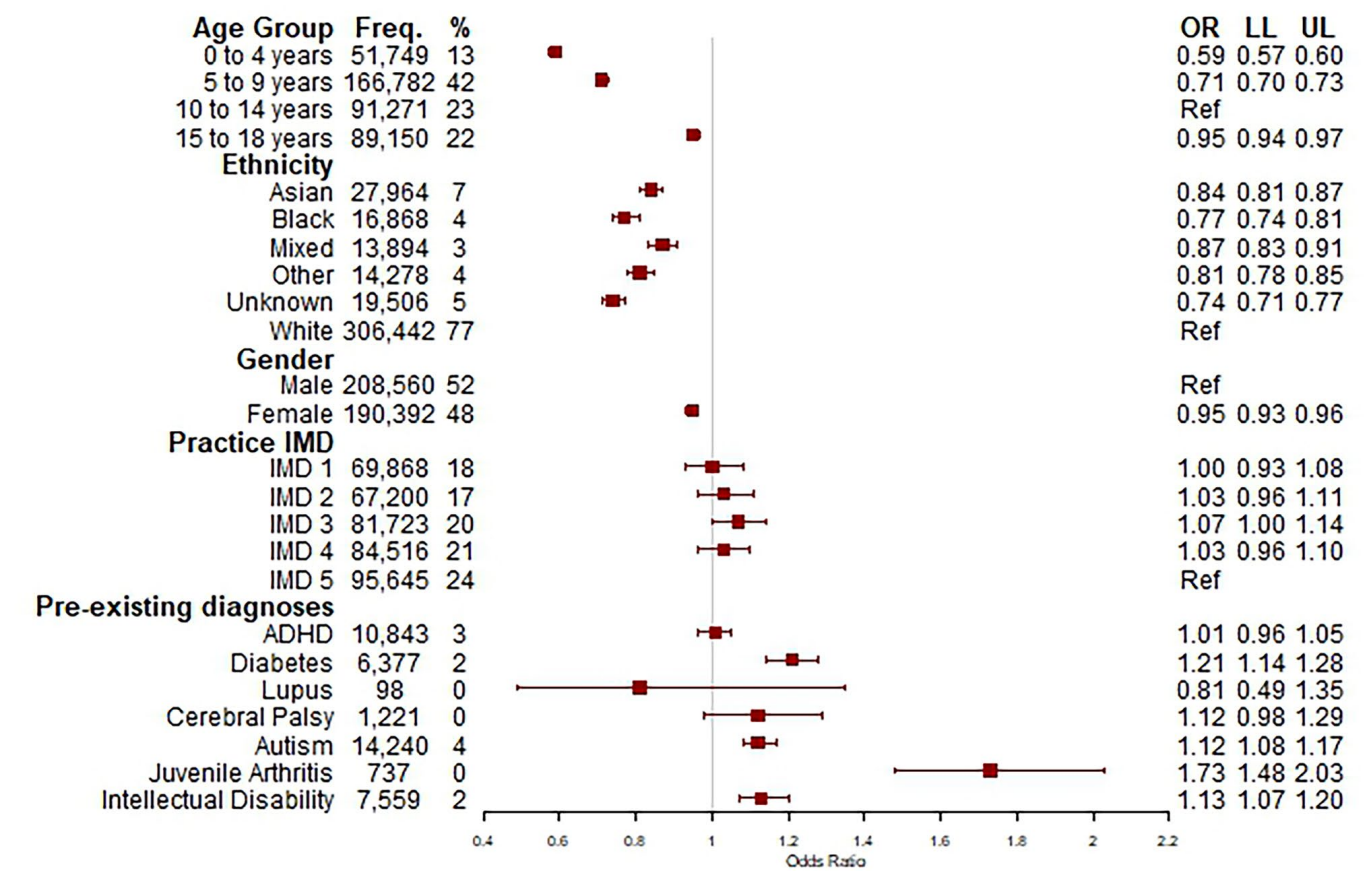
Logistic regression model of variables associated with the outcome of repeat consultations for all foot and ankle health encounters within six months during the study period

There were 83,197 (21%) out of 398,952 with repeat consultations for foot and ankle problems within 6 months (Fig. 2). Those in black, Asian and other ethnic groups had lower odds of repeat consultations compared to those in the white group, as did females compared to males (odds ratio 0.95, 95% confidence interval 0.93 to 0.96) (Fig. 2). There were increased odds of repeat consultations for CYP with pre-existing diagnoses: autism (1.12, 1.08 to 1.17), diabetes (1.21, 1.14 to 1.28), intellectual disabilities (1.13, 1.07 to 1.20) and juvenile arthritis (1.73, 1.48 to 2.03). The DAG is reported (online resources 3) and depicts assumed relationships between the exposure and outcome and all variables included in the fully adjusted analysis model. Factors associated with repeat consultations according to subgroups of code categories of the index consultation are reported (see online resources 4–7).

The average general practice in our sample had approximately 10,000 patients and 3500 patients aged 18 or younger (Table 4). In 1 year, such a general practice could expect to see 103 CYP (95% CI 83 to 122) with a first consultation for foot and ankle health, 41 patients (95% CI 28 to 53) in the musculoskeletal category, 21 dermatological (95% CI 12 to 30) and 25 unspecified pain (95% CI 15 to 35).

**Table 4:** Numbers of consultations and repeat consultations for foot and ankle diagnoses and diagnosis categories in a general practice with 10,000 patients

**Measures**	No index consultations per year expected in general practice with 3500 CYP patients*^a^
Total	103 (83 to 122)
Diagnosis category	
Musculoskeletal	41 (28 to 53)
Dermatological	21 (12 to 30)
Unspecified Pain	25 (15 to 35)
Infection	11 (5 to 18)
Fracture	8 (3 to 14)
Miscellaneous	4 (0 to 8)
Surgical	1 (− 1 to 2)
Nerve	0 (0 to 1)
Tumour	0 (0 to 0)
Circulatory	0 (0 to 0)
Diagnoses	
Ingrowing great toenail	15 (8 to 23)
Foot pain	12 (5 to 19)
Paronychia of toe	7 (2 to 12)
Ankle sprain	10 (4 to 16)
Ankle pain	7 (2 to 12)
Ankle injury	6 (1 to 10)
Foot injury	5 (1 to 10)
Injury of toe	3 (0 to 7)
Heel pain	3 (0 to 6)
Flat foot	4 (0 to 7)

*During study period 2015 to 2019 (COVID-19 pandemic years excluded)

^a^Number of CYP in the average general practice of 10,000 patients

## Discussion

This population-based cohort study is the largest analysis of foot and ankle problems in CYP to date. Among the 416,137 CYP with 687,753 coded events for foot and ankle problems, over a third of diagnoses were of musculoskeletal origin. The average rate of foot and ankle health consultations across the study period was 343 per 10,000 patient-years, peaking at 601 consultations per 10,000 patient-years among males aged 10 to 14 years in 2018.

Our data offers a broad analysis of reasons for GP consultation(s) and identified unspecified pain and dermatological conditions as common reasons for primary care consultation. Musculoskeletal diagnoses were the most common foot and ankle concerns in our cohort, and this echoes findings from a UK analysis of musculoskeletal problems in general practice [5]. We identified that children aged 10–14 years had the highest rates of consultation, and this is in line with previous work [5]. Whilst it is likely that rapid growth and skeletal changes are contributory factors [23], there is scope for further research to elucidate the sociodemographic and psychosocial factors, and mechanisms underpinning these problems, to inform the development of targeted clinical interventions.

We did not find associations with repeat consultations and practice IMD. Several sociodemographic and medical characteristics have been associated with frequent attendance in general practice in children [24], but there is little evidence documenting factors specific to foot and ankle problems. In an analysis of CPRD data for foot and ankle pain across the lifespan [1], there was no specific pattern for foot and/or ankle pain and socioeconomic group, whereas in an analysis of Australian data, children from deprived areas had a higher GP management rate of these conditions [2]. Our analysis did identify that CYP from minoritised ethnic groups had lower odds of repeat consultations compared to the white group, which corresponds with a recent scoping review identifying ethnic differences in access to a range of healthcare services [25]. Further in-depth qualitative work is recommended to explore this. Our findings also demonstrated that children with pre-existing diagnoses had higher odds of repeat consultations within 6 months which may be indicative of higher need among these groups. Medical characteristics have been associated with more frequent attendance, but these findings might reflect better engagement with services. There appears to be regional variation in the rate of consultations with London having much lower rates than the South East of England. As expected, there were lower rates for consultations during the COVID-19 pandemic, and this concurs with literature demonstrating lower health service attendance during this time [26]. Further research is required to understand whether access has returned to pre-pandemic levels and the effect on health inequalities. Evidence from NHS England indicates ongoing backlogs in care, particularly for CYP requiring community services such as physiotherapy [27].

### Strengths and limitations

A key strength of this study is the high-quality data [14], drawn from a large, longitudinal database, enabling the description of trends over time. The CPRD has high overall validity [14] but has not been investigated for foot and ankle problems specifically. The study population was selected using a list of codes established in previous research [1] and further refined with the input of clinical experts. Codes were categorised to highlight the clinical relevance of the descriptive findings and exploratory analyses. However, where codes were generic or ambiguous, it is likely that categories could overlap, for example, “ankle pain”, “ankle sprain” and “ankle swelling” were in different categories but could be equivalent diagnoses. There were missing data for covariates, in particular, BMI, where, due to the age of the population, only the BMI scores within the year of diagnoses were considered. A previous study indicated the potential for CPRD to underestimate the burden of foot and ankle health issues where chronic conditions were not recorded after the initial visit [1]. This may have led to the underestimation of repeat consultations in our study, particularly in the analysis of musculoskeletal consultations. Studies using CPRD data across a range of health conditions indicate that the completeness of data recording can be enhanced through consideration of linked data [28].

## Conclusion

The data reported in this study outlines the breadth of foot and ankle problems among CYP attending general practice. Our findings have identified that musculoskeletal and unspecified pain are the most common diagnosis category encountered by general practitioners. Given the complexity of some of these problems and the potential burden of repeat consultations, we recommend greater integration between general practice and services provided by allied health professionals such as podiatrists and physiotherapists, for example, through the Network Contract Directed Enhanced Service Additional Roles Reimbursement Scheme [29]. Further research is required to understand the reasons for regional and sociodemographic variation.

### Supplementary Information

Below is the link to the electronic supplementary material.

Supplementary file1 (DOCX 290 KB)

## Data Availability

The study is based on data from the Clinical Practice Research Datalink (CPRD) obtained under license from the UK Medicines and Healthcare Products Regulatory Agency (MHRA); however, the interpretation and conclusions contained in this report are those of the authors alone.

## Abbreviations

BMI: Body mass index
CI: Confidence intervals
CYP: Children and young people
CPRD: Clinical Practice Research Datalink
DAG: Directed acyclic graph
IRR: Incident rate ratios
ARR: Adjusted rate ratios
LL: Lower limit
UL: Upper limit

## Declarations

### Disclaimer

The views expressed are those of the author(s) and not necessarily those of the NIHR or the Department of Health and Social Care.
