# Supplementary material for: Foot and ankle problems in children and young people: a population-based cohort study
Source: Eur J Pediatr. 2024 May 9;183(8):3299–307. doi: 10.1007/s00431-024-05590-8 (PMC11263380; doi:10.1007/s00431-024-05590-8)
Supplement: Supplementary file 1 — Supplementary file1 (DOCX 290 KB) [file 431_2024_5590_MOESM1_ESM.docx]

**Online Resources: Foot and ankle problems in children and young people: a population-based cohort study.**

**Emma Rezel-Potts^1^, Catherine Bowen^2^, Kate M. Dunn^3^, Christopher I. Jones^4^, Martin C. Gulliford^1^, Stewart C. Morrison^1^**

**^1^School of Life Course and Population Sciences, King’s College London, Guy’s Campus, London SE1 1UL, UK.**

**^2^ Faculty of Environmental and Life Sciences, University of Southampton, Highfield Campus, SO17 1BJ, UK.**

**^3^ Centre for Musculoskeletal Health Research, School of Medicine, Keele University, Keele, Staffordshire, ST5 5BG, UK.**

**^4^ Department of Primary Care and Public Health, Brighton and Sussex Medical School, Falmer, BN1 9PS, UK.**

**Corresponding author**

**Dr Stewart Morrison, Addison House, Guy’s Campus, King’s College London,**

**London SE1 1UL, UK. Email:** [**stewart.morrison@kcl.ac.uk**](mailto:stewart.morrison@kcl.ac.uk)

Online Resource 2 Patient enrolment flow chart.

Total patients with foot and ankle coded events 1st January 2015 to 31st December 2021.

(N = 416,137)

Total patients aged ≤18 years and registered in English practices 1st January 2015 to 31st December 2021.

(N = 7,612,087)

Total patients available in CPRD Aurum source population and registered in practices actively contributing to the May 2022 Aurum release.

(N = 13,300,067)

All patients registered with the National Health Service (NHS).

Flow diagram representing the population and the sample drawn from this.

Online resource 3 Frequency of the 10 most commonly recorded foot and ankle consultation codes by gender.

| **Code Description** | **Frequency (%)** |
| --- | --- |
| **Males** |  |
| Total codes |  |
| Ingrowing great toenail | 68,971 (19) |
| Foot pain | 31,337 (8) |
| Paronychia of toe | 26,373 (7) |
| Ankle sprain | 19,852 (5) |
| Ankle injury | 17,467 (5) |
| Ankle pain | 17,074 (5) |
| Foot injury | 13,729 (4) |
| Heel pain | 11,355 (3) |
| Injury of toe | 11,246 (3) |
| Infection toe | 9,100 (2) |
| **Females** |  |
| **Total codes** |  |
| Ingrowing great toenail | 44,558 (13) |
| Foot pain | 36,239 (11) |
| Ankle sprain | 24,627 (7) |
| Ankle pain | 22,637 (7) |
| Ankle pain | 19,668 (6) |
| Paronychia of toe | 18,065 (5) |
| Ankle injury | 12,411 (4) |
| Foot injury | 9,120 (3) |
| Injury of toe | 7,754 (2) |
| Toe pain | 7,748 (2) |

The most commonly encountered consultation codes for boys and girls.

Online Resource 3 Directed acyclic graph (DAG) depicting assumed relationships


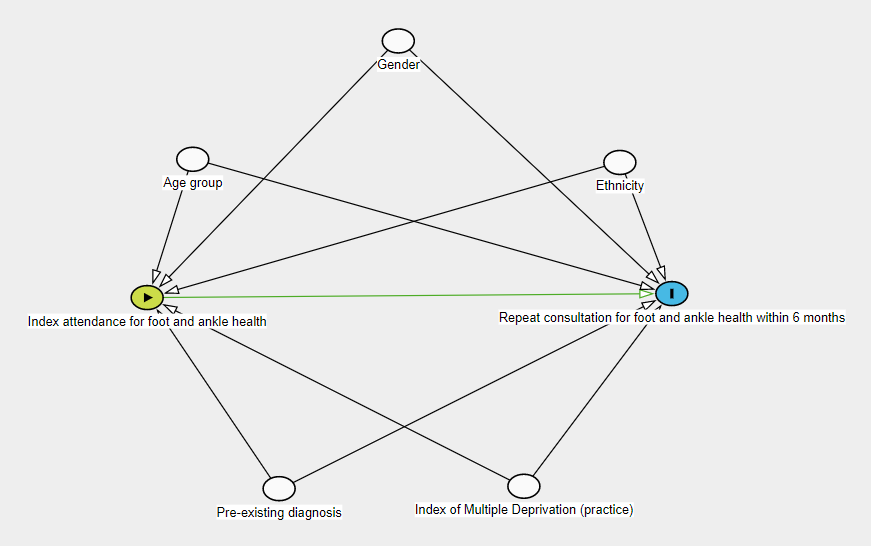


The DAG depicts assumed relationships between the exposure and outcome and all variables included in the fully adjusted analysis model.

Online resource 4 Repeat visits for musculoskeletal foot and ankle problems within 6 months


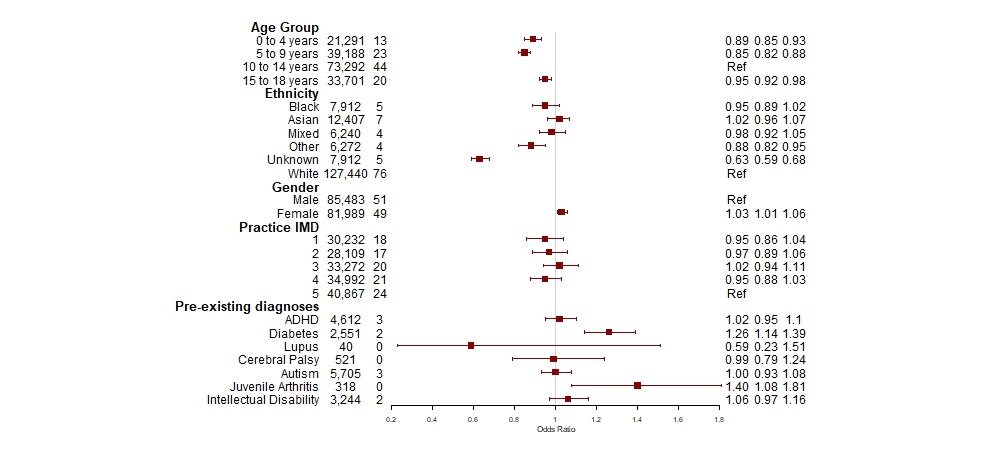


Logistic regression model of variables associated with the outcome of repeat visits for musculoskeletal foot and ankle health encounters within 6 months during the study period (n = 167,472).

Online resource 5 Repeat visits for dermatological foot and ankle problems within 6 months


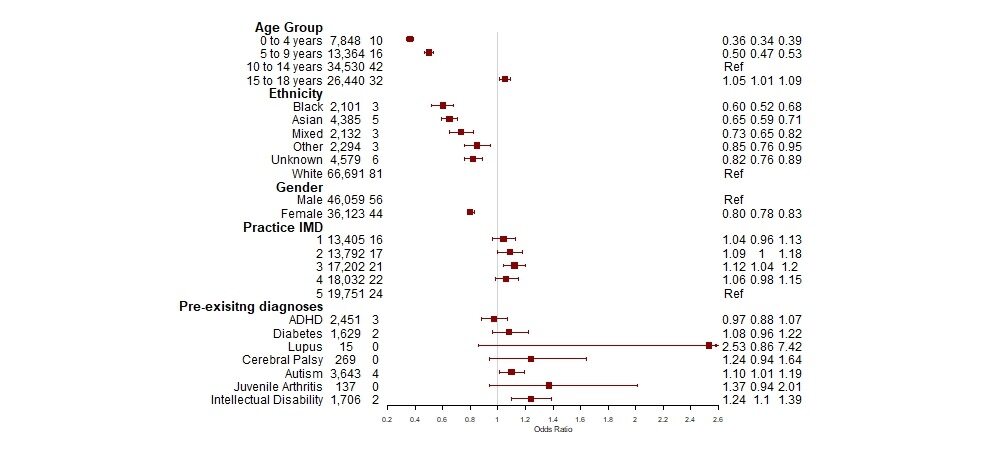


Logistic regression model of variables associated with the outcome of repeat visits for dermatological foot and ankle health encounters within 6 months during the study period (n=82,182).

Online resource 6 Repeat visits for unspecified foot and ankle problems within 6 months


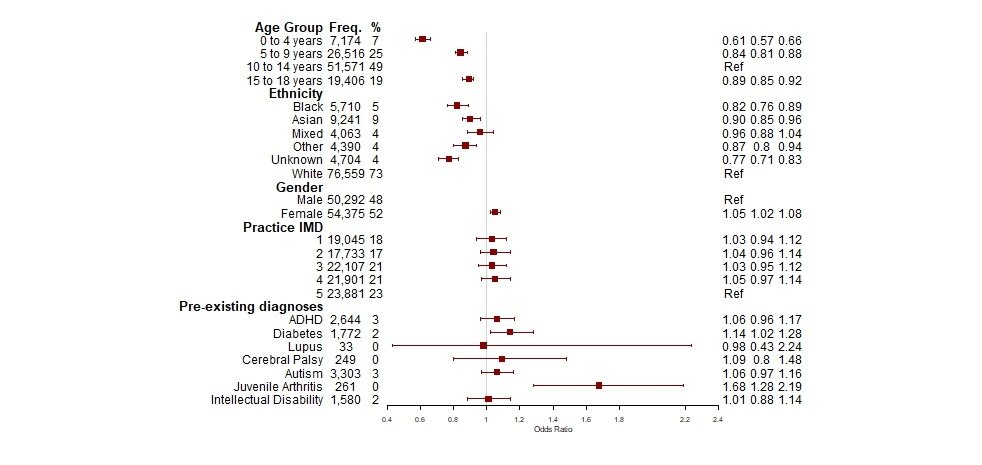


Logistic regression model of variables associated with the outcome of repeat visits for unspecified pain foot and ankle health encounters within 6 months during the study period, (n = 104,667).

Online resource 7 Repeat visits for foot and ankle infection within 6 months


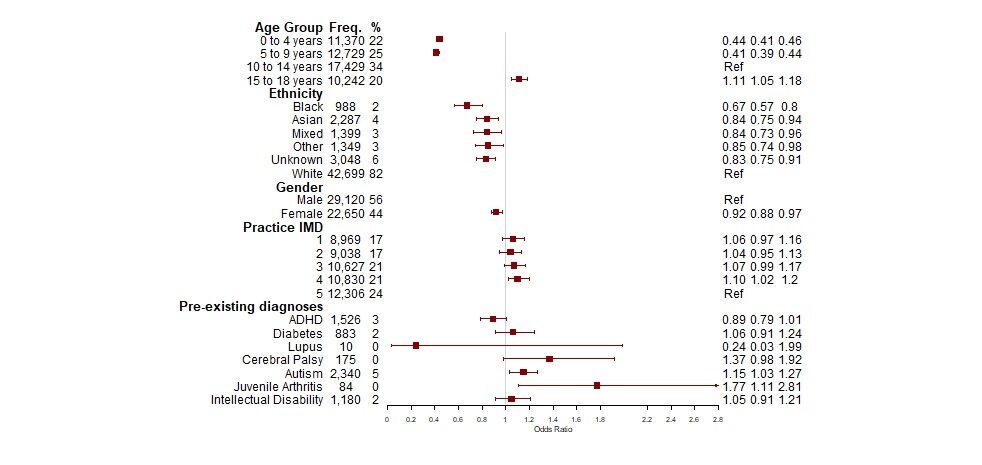


Logistic regression model of variables associated with the outcome of repeat visits for foot and ankle infection within 6 months during the study period (n = 51,770).
